# Comparing the effectiveness of universal admission testing and risk-based testing at emergency admission for preventing nosocomial COVID-19: a multicenter retrospective cohort study in Japan

**DOI:** 10.1017/ice.2024.161

**Published:** 2025-01

**Authors:** Kenta Iijima, Hitomi Osako, Kentaro Iwata

**Affiliations:** 1 Department of Infectious Disease and General Internal Medicine, Hyogo Prefectural Amagasaki General Medical Center, Amagasaki, Japan; 2 Department of Infection Control, Hyogo Prefectural Amagasaki General Medical Center, Amagasaki, Japan; 3 Division of Infectious Disease, Kobe University Hospital, Kobe, Japan

## Abstract

**Objective:**

To compare the effectiveness of universal admission testing (UAT) and risk-based testing (RBT) in preventing nosocomial coronavirus disease 2019 (COVID-19) after the implementation of strict infection control measures.

**Design:**

Retrospective multicenter cohort study.

**Setting:**

Five community hospitals in Japan.

**Patients:**

14,028 adult patients admitted emergently from June 1, 2022, to January 31, 2023.

**Methods:**

We calculated crude incidence density rates of community-acquired COVID-19 (positive test ≤4 days postadmission), hospital-acquired COVID-19 (positive test ≥8 days postadmission), total postadmission COVID-19 (all cases of positive test postadmission), and primary cases (sporadic and index cases). A generalized estimating equation model was used to adjust for local incidence (new COVID-19 patients per 100,000 population), single-bed room proportion, and admission proportion of patients older than 65 years.

**Results:**

The weekly local incidence in the study areas was less than 1,800 per 100,000 population (1.8%). Two hospitals implemented RBT and 3 implemented UAT. The median admission testing rate was higher in the UAT group than in the RBT group (95% vs 55%; difference 45.2%, 95% CI, 40.3%–48.8%). Crude and adjusted analyses revealed no significant associations between incidence density rates (IRR; >1 indicates higher incidence with UAT) and admission strategies for any of the outcomes: community-acquired cases (adjusted IRR = 1.23; 95% CI, 0.46–3.31), hospital-acquired cases (1.46; 0.80–2.66), total postadmission COVID-19 (1.22; 0.79–1.87), and primary cases (0.81; 0.59–1.12).

**Conclusions:**

Compared with risk-based testing, universal admission testing may have limited additional benefits in preventing nosocomial COVID-19 transmission during a period of low-moderate local incidence.

## Introduction

Compared with previous strains, the Omicron variant of severe acute respiratory coronavirus virus 2 (SARS-CoV-2) is more likely to cause asymptomatic or presymptomatic cases, with a model-estimated nosocomial infection rate of 3.4%, even when the local prevalence is below 1%.^1,2^ Although mortality is likely lower than that associated with earlier strains, nosocomial COVID-19 still poses significant problems, such as prolonged hospital stays due to delayed treatment and discharge.^2,3^ One measure against nosocomial COVID-19 is universal admission testing (UAT) of all patients for SARS-CoV-2.^4,5^

However, the additional benefits of UAT remain debated, especially with effective infection control measures like personal protective equipment and immunity from vaccination or past infection.^6^ In England and Scotland, discontinuing UAT was associated with 26%–41% increases in hospital-onset COVID-19.^5^ In contrast, a Japanese tertiary-care center did not observe increased nosocomial clusters after discontinuing UAT.^7^ Moreover, UAT has drawbacks, such as labor burdens, higher costs, and unnecessary procedures caused by false positives or results from prior infections.^8^

As an alternative to UAT, a risk-based testing (RBT) strategy examines only individuals with any COVID-19 symptoms, known exposure, or radiological findings suggestive of COVID-19.^9,10^ A modeling study showed that UAT was the most efficient approach when the local prevalence exceeded 4%, while testing only symptomatic cases was the most efficient otherwise.^1^ However, there is insufficient research directly comparing the effectiveness of UAT and RBT in preventing nosocomial COVID-19 and clusters in clinical practice, especially when considering local epidemic situations.

We hypothesized that UAT’s additional effectiveness might be limited when basic infection control measures are in place, and local incidence is moderate. Because secondary attack rates are lower in asymptomatic cases, clinical focus likely shifts to identifying symptomatic cases for early isolation.^11^ This study compares nosocomial COVID-19 rates between the UAT and RBT strategies among emergency hospitalized patients with high vaccine coverage during the Omicron era, providing insights for future infectious disease management.

## Method

### Study design, setting, and data collection

We conducted a cohort study comparing UAT and RBT strategies for preventing nosocomial COVID-19 after emergency admissions during the COVID-19 pandemic, driven by the Omicron variant in Japan.^12,13^ We retrospectively collected data from June 1, 2022, to January 31, 2023, at 5 voluntarily participating prefectural hospitals in Hyogo (Amagasaki General Medical Center, Awaji Medical Center, Harima-Himeji General Medical Center, Kakogawa Medical Center, and Tamba Medical Center). All hospitals were designated regional medical care support hospitals, providing regional medical care and support for family doctors.^14^ Data were obtained from the Diagnosis Procedure Combination (DPC) database (age, sex, admission and discharge dates, principal diagnoses, or triggers for hospitalization) and electronic medical records (SARS-CoV-2 examination dates and results). The DPC system standardizes electronic claims for inpatients in acute care hospitals in Japan.^15^ We calculated the crude incidence density rates of COVID-19 after admission to compare the UAT-adopted hospital group with the RBT-adopted group. Then, a generalized estimating equation model was used to adjust the incidence density rates with covariates. We documented the infection control measures policies of the participating hospitals, such as criteria defining close contact, isolation periods, staff work restrictions, visitation restrictions, and monthly alcohol-based hand rub consumption data in general wards.^16^ City offices in the participating hospitals’ cities provided vaccination rates, calculated using the number of vaccinated individuals and city population from the Basic Resident Registration.^17^

This study followed the Strengthening the Reporting of Observational Studies in Epidemiology (STROBE) reporting guideline.^18^

### Participants

Participants included all patients aged 18 years or older who were emergently admitted and discharged during the study. We followed patients for SARS-CoV-2 test results and readmissions within 1 week of discharge. We excluded patients with scheduled admissions or patients aged 17 or younger, as no pediatric department adopted the UAT strategy. We also excluded patients who were previously diagnosed as having COVID-19 within the past 30 days by healthcare services, hereafter referred to as previously diagnosed COVID-19, as they usually did not require screening testing and were managed in separate wards.

### Variable

#### Admission test strategies

In hospitals adopting the UAT strategy, all emergency-admitted patients underwent antigen or polymerase chain reaction (PCR) tests for SARS-CoV-2. The RBT strategy restricted testing to patients with symptoms, exposure history, or findings indicative of pneumonia. We defined admission tests as tests performed from 4 days before admission to on admission day. The cutoff was based on the detectability of Omicron strains up to 4 days before symptom onset.^19–21^ Neither strategy tested patients with a history of COVID-19 within the past 3 months. We calculated the testing rates and medians for each hospital week by dividing the number of tests on admission by the number of emergency admissions in each strategy group, excluding previously diagnosed COVID-19. The 95% confidence intervals for the medians were calculated using the bootstrap method.

#### Case definitions

Patients with a positive antigen or PCR result for SARS-CoV-2 were categorized by the date of the first positive test. A positive COVID-19 test at admission classified the case as a COVID-19 admission. Patients who tested positive from the day after admission to 4 days after discharge were classified as postadmission COVID-19. The infection control teams (ICTs) in each hospital reviewed the electronic medical records of postadmission COVID-19 and cases coded as the ICD-10 code for COVID-19 (U07.1) without laboratory-confirmed positive results to identify previously diagnosed COVID-19. Noninfectious cases were determined as asymptomatic individuals with documented COVID-19 10 days to 3 months prior or, in the case of PCR testing, with a Ct value of ≥30.

Postadmission COVID-19 was further categorized as follows: positive tests from day 1 to day 4 of hospitalization were considered community-acquired, day 5 to day 7 as indeterminate, and after day 7 as hospital-acquired. The ICTs determined whether postadmission COVID-19 corresponded to a cluster based on the presence of 2 or more epidemiologically linked COVID-19 cases, including inpatients and healthcare workers.

We counted primary cases, which included index cases of nosocomial clusters and sporadic cases without epidemiological links, to examine the associations between admission testing and nosocomial clusters. These primary cases can be the first trigger of a nosocomial cluster.^22^

#### Outcome measure

We measured the weekly crude incidence density rates per 1,000 at-risk patient days for each hospital week for community-acquired, hospital-acquired, and primary cases. Additionally, we assessed the incidence of total postadmission COVID-19 (including community-acquired, hospital-acquired, and indeterminate cases) to evaluate the overall impact of admission testing, regardless of symptom onset timing. The at-risk patient days were calculated based on the length of stay (LOS) until the day the hospitalized patient tested positive for SARS-CoV-2. Previously diagnosed COVID-19 and COVID-19 admission patients were excluded from calculating all outcomes due to their management in separate COVID-19 wards, distinct from the general patient population.

### Covariates

#### Local COVID-19 incidence levels

We defined new case levels (NCLs) to indicate local COVID-19 incidence levels based on the definition proposed by the Centers for Disease Control and Prevention.^23^ The NCL was calculated as the 7-day total number of newly diagnosed COVID-19 patients per 100,000 population in each hospital’s city. We converted the NCL to a weekly local incidence rate expressed as a percentage. The number of new cases and the denominator population data were obtained from the Hyogo Prefectural Government^24^ and the latest census data in Japan in October 2020.^25^

#### Proportion of single-bed rooms

Managing patients in private rooms reduces the transmission of nosocomial infections.^26^ We calculated the proportion of single-bed rooms based on the number of single-bed rooms and the total number of hospital beds for each hospital.

#### Admission proportion of patients aged 65 or older

A higher proportion of older admitted people is associated with higher nosocomial infection rates.^27^ We calculated weekly the proportion of newly admitted patients aged 65 years or older who were eligible for emergency admissions, excluding previously diagnosed COVID-19.

### Statistical analysis

Descriptive statistics were used to describe the hospital and patient characteristics of the UAT and RBT groups. We used a generalized estimating equation (GEE) with a negative binomial distribution model to analyze the relationship between the incidence density rates of outcomes for each hospital week and the admission screening strategies, adjusting for covariates including NCL, the proportion of single-bed rooms, the admission proportion of patients aged 65 or older, and the week number. The incidence density rate ratio (IRR) and its 95% confidence intervals, calculated from the model coefficients, were used to evaluate the results. The 95% confidence interval was calculated as 1.96 times the standard error of the coefficient, and the IRR was obtained by exponentiating the coefficient. An autoregressive correlation matrix was used to account for the influence of patient readmissions and the epidemic situation of the previous week. The offset term contained in the model consists of the weekly at-risk patient days per 1,000.

As a sensitivity analysis, we compared each endpoint when changing the hospital-acquired cutoff from 7 to 4 days and the community-acquired cutoff from 4 to 7 days. A two-sided test with a significance level of *P* < 0.05 was used. The statistical software used was R (version 4.3.2) and RStudio (version 2023.06.0+421). For GEE, we used the “reticulate” package in R to utilize “statsmodels” in Python (version 3.10.5).

### Ethics approval

This study was approved by the local ethics committee at Amagasaki General Medical Center (approval no. 5–45).

## Results

Characteristics of hospitals and patients are described in Table 1. Three hospitals adopted the UAT strategy, and 2 adopted the RBT strategy. The number of beds, the proportion of single-bed rooms, and alcohol-based hand rub consumption were comparable between the 2 groups. The vaccination rates among citizens were generally consistent between the 2 groups and across the participating hospitals’ cities (Table 1, Table S1). Each hospital had similar strict standards for preventing nosocomial infections, including visiting restrictions, personal protective equipment usage, and isolation periods for patients and staff (Table S1).

**Table 1.** Characteristics of the 5 hospitals and patients who underwent screening via each testing method, excluding COVID-19 admission patients

Characteristics	Overall	Universal admission testing	Risk-based testing	*P* value
Hospital, N	5	3	2	–
Beds, mean (SD)*	496.60 (180.44)	437.33 (176.24)	585.50 (204.35)	.447
Proportion of single-bed rooms, mean (SD)*	0.31 (0.10)	0.37 (0.08)	0.23 (0.05)	.106
Alcohol-based hand rub consumption per month, mean (SD)*	17.48 (7.79)	15.76 (9.14)	20.06 (7.32)	.621
Vaccination rates of citizens in cities with participating hospitals				
Two vaccine doses, median [IQR]^†^	0.82 [0.79–0.82]	0.82 [0.80–0.82]	0.81 [0.79–0.82]	1
Citizens aged 65 or over with 2 doses, median [IQR]^†^	0.94 [0.94–0.94]	0.94 [0.94–0.94]	0.93 [0.93–0.94]	.248
Three vaccine doses, median [IQR]^†^	0.56 [0.55–0.65]	0.56 [0.55–0.62]	0.60 [0.58–0.63]	1
Citizens aged 65 or over with 3 doses, median [IQR]^†^	0.89 [0.89–0.89]	0.89 [0.89–0.90]	0.88 [0.88–0.89]	.248
Screened patients, N	14,028	7,092	6,936	
Age, median [IQR]^†^	74.0 [57.0–83.0]	75.0 [61.0–84.0]	73.0 [52.0–82.0]	<.01
Patients aged 65 or over^‡^	9,430 (67.2)	5,021 (70.8)	4,409 (63.6)	<.01
Women^c^	6,735 (48.0)	3,200 (45.1)	3,535 (51.0)	<.01
Length of hospital stay days, median [IQR]^†^	10 [6–19]	11 [6–19]	10 [6–19]	.7
Admission without testing^‡^	3,940 (28.1)	446 (6.3)	3,494 (50.4)	<.01
Admission testing reagents^‡^				<.01
Antigen	2,831 (28.1)	925 (13.9)	1,906 (55.4)	–
Polymerase chain reaction	7,257 (71.9)	5,721 (86.1)	1,536 (44.6)	–
Total postadmission COVID-19^‡^	146 (1.0)	81 (1.1)	65 (0.9)	.27
Overall non-COVID-19 patients days	–	99,703	100,920	–
Total postadmission COVID-19	N=146 (100%)	N=81 (100%)	N=65 (100%)	–
Categorization by timing of onset				
Community-acquired^‡^	26 (17.8)	10 (12.3)	16 (24.6)	.09
Indeterminate^‡^	10 (6.8)	6 (7.4)	4 (6.2)	1
Hospital-acquired^‡^	110 (75.3)	65 (80.2)	45 (69.2)	.18
Classification by cluster formation				
Patients involved in clusters^‡^	99 (67.8)	44 (67.7)	55 (67.9)	1
Sporadic onset cases^‡^	47 (32.2)	21 (32.3)	26 (32.1)	1
Primary cases	91	46	45	–
Number of clusters, N	44	20	24	–
Days to onset of symptoms after admission (median [IQR])^†^	13.5 [8.0–22.0]	15.0 [8.0–24.0]	12.0 [6.0–18.0]	.17
Testing on admission among nosocomial onset cases				
Negative results^‡^	114 (78.1)	80 (98.8)	34 (52.3)	<.01
Not tested^‡^	32 (21.9)	1 (1.2)	31 (47.7)	<.01

Note. COVID-19, coronavirus disease 2019; SD, standard deviation; IQR, interquartile range.

Data are presented as N (%) otherwise indicated.

*Independent *t* test was used for normally distributed data.

^†^Mann‒Whitney *U* test was used for nonnormally distributed data.

^‡^χ^2^ test used in categorical variables.

**Figure 1.**
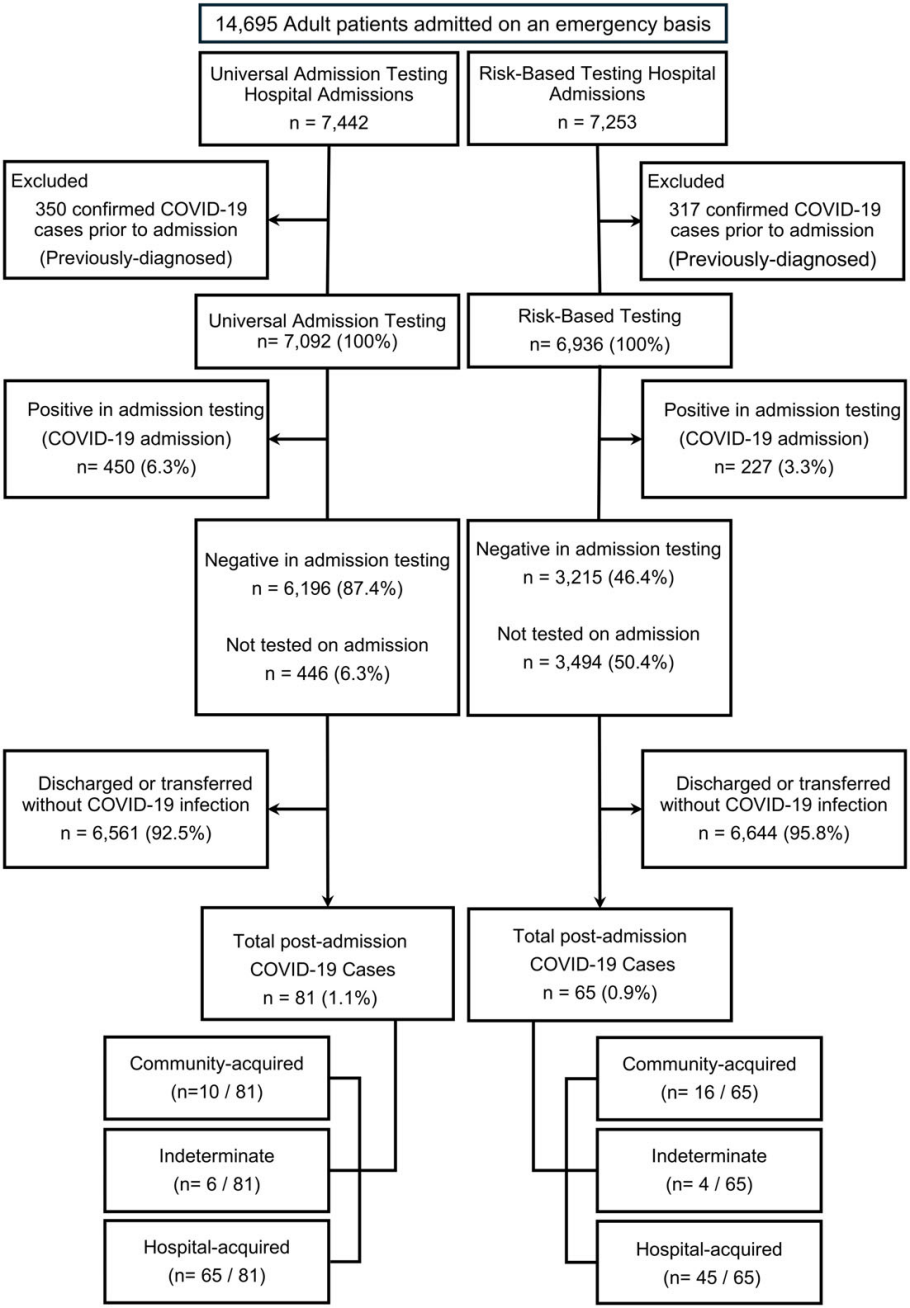
Flow diagram of patient enrollment in the study.

From June 1, 2022, to January 31, 2023, we included 14,695 adults who were emergently admitted to the 5 hospitals. We excluded 667 patients who had been diagnosed as having COVID-19 at a previous hospital or in the outpatient department before admission. We measured the outcomes of 14,028 patients who underwent screening with each testing method, excluding 677 COVID-19 admission patients (Table 1, Figure 1). The median age was 74 years (IQR 57–83 years), 6,735 (48%) were women, and the median LOS was 10 days (IQR 6–19 days). Patients in the UAT group were older and had lower proportions of women, admissions without testing (6.3% vs 50.4%), and antigen tests used for admission (13.9% vs 24.8%) than patients in the RBT group did (all *P* < 0.01; Table 1). We confirmed postadmission COVID-19 in 81 (1.1%) patients in the UAT group and 65 (0.9%) patients in the RBT group.

**Figure 2.**
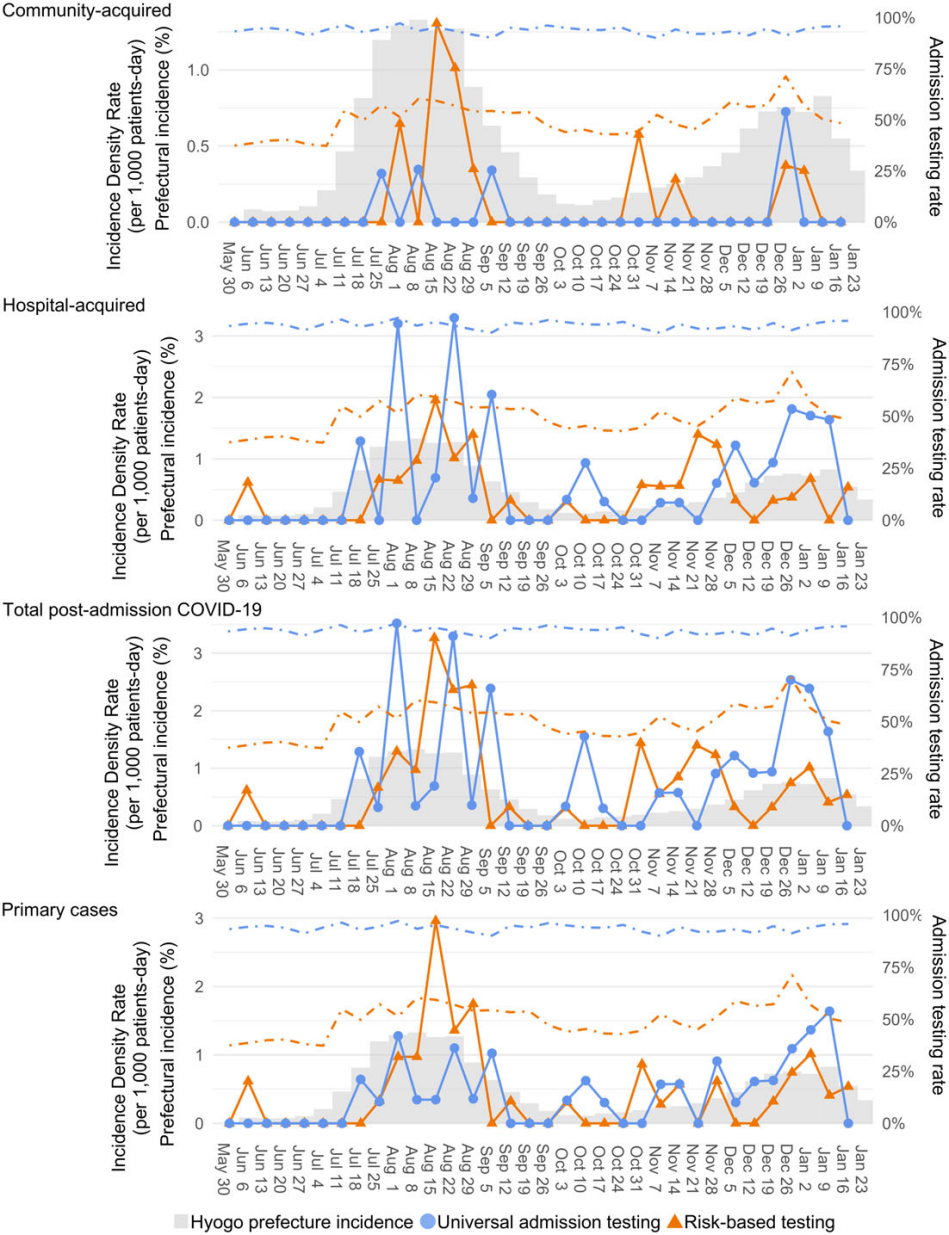
Crude weekly mean incidence density rate (solid line), admission testing rate (dashed line), and local incidence rate in Hyogo Prefecture (bar graph). Universal admission testing group (filled circle, blue) and risk-based testing group (filled triangle, orange). The vertical axis shows (left-most) the incidence density rate per 1,000 at-risk patient days, (left-most) Hyogo Prefecture’s incidence rate (%), and (right-most) the testing rate (%).

During the study period, the maximum weekly local incidence of COVID-19 was 1.7% from August 3 to August 9, 2022 (Figure S1). We obtained 175 hospital weeks from 5 distinct hospitals, which reported data for all 35 weeks of the study period (Table 2). The median admission testing rate in the UAT group was greater than that in the RBT group (95% vs 55%; *P* < 0.01), and the difference was 45.2% (95% CI, 40.3%–48.8%). In addition, the proportion of patients aged 65 years or older in the UAT group was greater than that in the RBT group (72% vs 64%; *P* < 0.01). The crude weekly incidence density rates and admission testing rates are described in Figure 2.

**Table 2.** Crude median weekly admission screening results based on hospital-week data

Weekly admission screening	Overall	Universal admission testing	Risk-based testing	*P* value
Hospital-week	N=175	N=105	N=70	
Local COVID-19 incidence levels (new case levels)	333.75 [145.28–663.52]	338.34 [177.38–677.67]	317.26 [134.66–609.18]	.439
Admission testing rate	0.89 [0.57–0.96]	0.95 [0.91–0.98]	0.55 [0.43–0.62]	<.001
Difference of median testing rate, % (95% CI)	–	45.2 (40.3–48.8)	Reference	–
Proportion of patients aged 65 or older	0.70 [0.62–0.75]	0.72 [0.67–0.75]	0.64 [0.58–0.75]	<.001
Previously diagnosed COVID-19 cases, N	2 [1–5]	2 [0–4]	2 [1–6.75]	.107
New admission cases, N	66 [54.50–108.50]	58 [50–95]	82 [66–139.25]	<.001
New admission cases of COVID-19 admission, N	3 [1–5.50]	3 [1–6]	2 [1–4]	.074
Not tested new admission cases, N	7 [3–29]	4 [2–6]	39 [24.25–78]	<.001
New admission cases with negative results on testing, N	47 [39–59]	49 [41–85]	44.50 [38–55.75]	.015
Non-COVID-19 inpatients cases, N	214 [141–327.50]	153 [127–304]	243 [219.25–383.50]	<.001
Weekly at-risk patient days	1,065 [707–1671.50]	753 [632–1549]	1,301 [1,168.75–1,895.75]	<.001
Crude weekly incidence density rate, mean (SD)*				
Community-acquired, mean (SD)*	0.09 (0.33)	0.06 (0.31)	0.12 (0.35)	.229
Hospital-acquired, mean (SD)*	0.59 (1.35)	0.68 (1.63)	0.45 (0.78)	.267
Total postadmission COVID-19	0.79 (1.82)	0.91 (2.20)	0.62 (1.02)	.303
Primary cases	0.52 (1.43)	0.57 (1.73)	0.44 (0.79)	.548

Note. COVID-19, coronavirus disease 2019; CI, confidence interval; SD, standard deviation.

Table 3 summarizes the crude and adjusted associations of the admission testing strategy with each outcome. In the crude analysis, the incidence density rates of all outcomes were not significantly associated with the admission strategies. The adjusted analysis with GEE models showed that none of the incidence density rates of each outcome were significantly associated with admission strategies. Although the difference was not significant, the UAT group tended to have fewer primary cases (adjusted IRR = 0.81; 95% CI, 0.59–1.12, Forest plot in Figure S2).

**Table 3.** Crude and adjusted weekly mean incidence density rate and incidence rate ratio

Outcomes	Mean incidence density rate (95% CI)		*P* value	Incidence rate ratio (95% CI)	
	Universal admission testing	Risk-based testing		Crude	Adjusted
Community-acquired	0.06 (0.00–0.63)	0.12 (0.00–1.20)	0.10	1.98 (0.62–6.34)	1.23 (0.46–3.31)
Hospital-acquired	0.68 (0.00–4.81)	0.45 (0.00–2.43)	0.55	0.66 (0.36–1.21)	1.46 (0.80–2.66)
Total postadmission COVID-19	0.91 (0.00–6.05)	0.62 (0.00–3.57)	0.54	0.68 (0.37–1.24)	1.22 (0.79–1.87)
Primary cases	0.57 (0.00–2.74)	0.44 (0.00–2.74)	0.63	0.77 (0.38–1.57)	0.81 (0.59–1.12)

Note. CI, confidence interval; COVID-19, coronavirus disease 2019.

Mann‒Whitney *U* test was used for nonnormally. Data are presented as median (IQR, interquartile range) and analyzed using the Mann‒Whitney *U* test for nonnormally distributed data unless otherwise indicated.

* Independent *t* test was used for normally distributed data.

Similarly, even after changing the hospital-acquired cutoff from 7 to 4 days and the community-acquired cutoff from 4 to 7 days, the sensitivity analyses also demonstrated consistent results (Figure S3).

## Discussion

In this retrospective multicenter cohort study, UAT was not associated with a significant reduction in any outcome, including community-acquired, hospital-acquired, or primary cases. These findings suggest that the impact of UAT on reducing nosocomial COVID-19 is very limited during periods of moderate local incidence less than 1.8%, despite an increase in testing rate by a median of 45.2% (95% CI, 40.3%–48.8%).

A computational modeling study using English acute hospitals data estimated that UAT could reduce nosocomial COVID-19 incidence by 8.1%–21.5%, with a more pronounced effect when the prevalence exceeded 2%.^1^ Importantly, this model found testing only symptomatic cases at admission was the most efficient strategy for detecting cases per test, regardless of prevalence.^1^ Our results support this model’s findings, suggesting that when the incidence is not high, UAT may detect a few additional cases potentially causing nosocomial transmission. However, our results differ from an interrupted time series analysis across multiple facilities in England and Scotland, which found that stopping UAT led to significant increases in hospital-acquired cases (41% in Scotland, 26% in England).^5^ This discrepancy might be attributed to the higher local incidence rates in the United Kingdom, which exceeded 1.9%.^28^ This underscores the importance of considering local epidemiology when interpreting COVID-19 testing strategies.

Notably, the UK study did not report changes in testing rates after UAT cessation, which could have affected their results. A United States multicenter retrospective cohort study revealed that when the monthly testing rate exceeded 50%, hospital-acquired cases were significantly lower than at a 25%–50% monthly rate (rate ratio 0.87; 95% CI, 0.78–0.98).^27^ Although this study adjusted for the positive-result testing rate instead of the local epidemic level, a testing rate below 50% might be insufficient to detect COVID-19 at admission with an incidence of at least 1.5% in the United States.^27,28^ Our RBT group maintained a high median testing rate of 55%, which falls into “the high testing stratum” in this US study.^27^ Despite this high testing rate, the approximately 50% reduction in testing compared to UAT still represents a significant improvement in resource utilization.

In our study, the lack of significant difference between UAT and RBT may be due to factors beyond moderate local incidence. Nosocomial spread may have been suppressed by measures such as patient isolation, proper use of personal protective equipment, strict visitor regulations, and universal masking, supported by high citizens’ vaccination rates (82%–94% with 2 doses, 56%–90% with 3 doses).^29^ In the RBT group, where the testing burden was reduced, hospitals may have compensated for fewer tests by strengthening surveillance for symptomatic cases in wards. This could have led to early isolation of primary cases, effectively interrupting subsequent nosocomial transmission. Our results suggest that prompt intervention can suppress hospital-acquired incidence, even if primary cases occur.

The strengths of this study include the direct comparison of hospital testing policies across multiple facilities during the same period. It improves upon previous studies by using NCL as an indicator of local incidence and considering the single-bed room proportion, crucial for infection control. Additionally, we separately evaluated the number of primary cases triggering clusters, as cluster occurrence complicates assessing the relationship between admission testing and outbreaks.

This study has several limitations. First, a significant limitation is the absence of data on postadmission testing policies across participating hospitals. Contrary to our previous argument, UAT hospitals might have conducted more aggressive surveillance, potentially leading to higher hospital-acquired incidence. Although we assume strict COVID-19 monitoring in both UAT and RBT hospitals given the pandemic context, we lack quantitative data, including tests per symptomatic event. This study did not perform universal discharge testing, raising concerns about insufficient tracking of asymptomatic nosocomial infections as only 30% (21%–40%) of nosocomial infections become symptomatic.^1,30^ However, the benefit of identifying asymptomatic postadmission COVID-19 is unclear because asymptomatic cases are not usually treated, repeated testing is extensive, and the viral load may not always be sufficient for detection at discharge.^3,26^ Second, our small sample size resulted in wide confidence intervals for our effect estimates, limiting the detection of potential associations and adjustment for hospital-level practice variations. Third, as a retrospective study, the influence of unknown confounding factors cannot be eliminated. The observed change in the IRR direction after adjustment warrants careful consideration, although these were not significant. This change reflects the greater proportion of single rooms than in the RBT group as we adjusted for, though other unmeasured confounders may have influenced it. To address this limitation, we have provided detailed descriptions of infection control practices for each hospital (Table S1). Although we could not adjust for all these factors in our statistical model due to the complexity and potential collinearity, we believe this information demonstrates that there were no major differences in infection control practices among the hospitals. Fourth, the UAT and RBT groups used different testing reagents. Given that antigen tests are 28%–41% less sensitive than PCR,^31^ this could have influenced the results. However, the UAT group used fewer antigen tests, making it unlikely that reagent variation underestimated UAT’s effects. Fifth, our results apply to adult emergency admissions in community hospitals. However, because community hospitals have a higher risk of community-acquired cases than tertiary-care, pediatric, and behavioral health hospitals, underestimation is unlikely.^32^ Finally, NCLs in our study may have been underestimated, especially among low-risk individuals with mild symptoms. Japan’s public reporting system likely ensured reliable case counts. However, our findings may not apply to high-prevalence scenarios where stricter measures, including universal testing, might be necessary. These limitations should be considered when interpreting our results.

In conclusion, our study showed that, compared with risk-based testing, universal admission testing offers limited additional benefits in preventing nosocomial COVID-19 transmission during low-moderate local incidence. We recommend that healthcare institutions and policymakers consider local epidemiology and resource constraints when determining testing strategies. Optimal allocation between admission testing and postadmission infection control measures is crucial for future SARS-CoV-2 pandemics and emerging infectious diseases.

## Supporting information

Iijima et al. supplementary material Iijima et al. supplementary material

## Supplementary material

To view supplementary material for this article, please visit https://doi.org/10.1017/ice.2024.161.
