# Supplementary material for: Comparing the effectiveness of universal admission testing and risk-based testing at emergency admission for preventing nosocomial COVID-19: a multicenter retrospective cohort study in Japan
Source: Infect Control Hosp Epidemiol. 2024 Oct 22;46(1):81–9. doi: 10.1017/ice.2024.161 (PMC11717481; doi:10.1017/ice.2024.161)
Supplement: Iijima et al. supplementary material [file S0899823X24001612sup001.docx]

**Supplementary**

**Comparing the Effectiveness of Universal Admission Testing and Risk-Based Testing at Emergency Admission for Preventing Nosocomial COVID-19: A Multicenter Retrospective Cohort Study in Japan**

**Running title:** Comparing admission testing for nosocomial COVID-19

Kenta Iijima MD ^a,c,*^, Hitomi Osako MN ^b^, Kentaro Iwata MD, PhD ^c^

**Affiliations**

^a^ Department of Infectious Disease and General Internal Medicine, Hyogo Prefectural Amagasaki General Medical Center, Amagasaki, Japan.

^b^ Department of Infection Control, Hyogo Prefectural Amagasaki General Medical Center, Amagasaki, Japan.

^c^ Division of Infectious Disease, Kobe University Hospital, Kobe, Japan.

***Corresponding author**. Address: Department of Infectious Disease and General Internal Medicine, Hyogo Prefectural Amagasaki General Medical Center, 2-17-77 Higashi Naniwa-Cho, Amagasaki,660-8550, Japan. Tel.: +816 6 480 7000.

E-mail address: kenta.iijim@gmail.com (Kenta Iijima).

**Supplementary**

**Table S1.** Hospitals, Vaccination Rates among Citizens in the City of Hospitals, and Infection Control Policies within Hospitals

**Figure A.** Line graph of the weekly local COVID-19 incidence in Hyogo Prefecture and the cities where the participating medical institutions are located during the study period.

**Figure B.** Forest plot of mean incidence rate ratio and 95% confidence intervals for each outcome.

**Figure C.** Forest plot of mean incidence rate ratio and 95% confidence intervals for each outcome, with changes to the cut-off for hospital-acquired and community-acquired cases.

**Table S1.** Hospitals, Vaccination Rates among Citizens in the City of Hospitals, and Infection Control Policies within Hospitals

| **Hospital** | **AGMC** | **AMC** | **HGMC** | **KMC** | **TMC** |
| --- | --- | --- | --- | --- | --- |
| Number of beds | 730 | 441 | 640 | 352 | 320 |
| Proportion of single-bed rooms ^*^ | 0.26 | 0.19 | 0.32 | 0.47 | 0.34 |
| City of hospital | Amagasaki | Awaji | Himeji | Kakogawa | Tamba |
| Vaccination rate among citizens (%) |  |  |  |  |  |
| One dose | 0.79 | 0.84 | 0.80 | 0.82 | 0.82 |
| Citizens aged 65 or over with one dose | 0.93 | 0.94 | 0.94 | 0.94 | 0.94 |
| Two doses | 0.78 | 0.83 | 0.79 | 0.82 | 0.82 |
| Citizens aged 65 or over with two doses | 0.93 | 0.94 | 0.94 | 0.94 | 0.94 |
| Three doses | 0.55 | 0.65 | 0.55 | 0.67 | 0.56 |
| Citizens aged 65 or over with three doses | 0.87 | 0.89 | 0.89 | 0.91 | 0.89 |
| **Infection control policies** |  |  |  |  |  |
| Admission testing strategy | Risk-Based Testing^†^ | Risk-Based Testing^†^ | Universal Admission Testing^‡^ | Universal Admission Testing | Universal Admission Testing^§^ |
| PCR reagents used | Film Array® (bioMérieux) AutoAmp Ampdirect™ 2019-nCoV Detection Kit (Shimadzu Corporation) | Film Array® | AutoAmp Ampdirect™ 2019-nCoV Detection Kit (Shimadzu Corporation) Cobas Liat SARS-CoV-2 & Flu A/B (Roche Diagnostics) | Cobas Liat SARS-CoV-2 & Flu A/B | Film Array® Loopamp SARS-CoV-2 Detection Kit(Eiken Chemical) |
| Antigen reagents used | Lumipulse SARS-CoV-2 Ag (Fujirebio) | ImmunoAce SARS-CoV-2 (TAUNS Laboratories) | No usage | No usage | Lumipulse SARS-CoV-2 Ag |
| Visiting restrictions | Prohibited | Prohibited | Prohibited | Prohibited | Prohibited |
| Universal masking | Both patients and healthcare workers wear surgical masks. | Both patients and healthcare workers wear surgical masks. | Both patients and healthcare workers wear surgical masks.  During aerosol procedures, healthcare workers were required to wear N95 masks until the patient's hospital stay exceeded 5 days. | Both patients and healthcare workers wear surgical masks.  During aerosol procedures, healthcare workers were required to wear N95 masks. | Both patients and healthcare workers wear surgical masks. |
| Universal masking during aerosol-generating procedures (AGPs) | N95 masks were used only during AGPs in the emergency department and for intubation and extubation during surgery. | N95 masks were used only during AGPs in the emergency department and for intubation and extubation during surgery. | N95 masks were used during AGPs until the patient's admission duration exceeded 5 days. | N95 masks were used only during AGPs for intubation and extubation in surgery. | N95 masks were used for AGPs only in confirmed or suspected COVID-19 cases. |
| Personal protective equipment (PPE) usage for confirmed or suspected COVID-19 patients | Entering a room with confirmed or suspected COVID-19 patients: goggles, surgical mask, glove, and long-sleeve gown.  During aerosol procedures, healthcare workers were required to wear N95 masks. | Entering a room with confirmed or suspected COVID-19 patients: cap, goggles, surgical mask, glove, and long-sleeve gown.  During aerosol procedures, healthcare workers were required to wear N95 masks. | Entering a room with confirmed or suspected COVID-19 patients: cap, goggles, surgical mask, glove, and long-sleeve gown.  During aerosol procedures, healthcare workers were required to wear N95 masks. | Entering a room with confirmed or suspected COVID-19 patients: cap, goggles, surgical mask, glove, and long-sleeve gown.  During aerosol procedures, healthcare workers were required to wear N95 masks. | Entering a room with confirmed or suspected COVID-19 patients: cap, goggles, surgical mask, glove, and long-sleeve gown.  During aerosol procedures, healthcare workers were required to wear N95 masks. |
| Patients isolation period | 10 days | 10 days | 10 days | 10 days | 10 days |
| Staff isolation period (Symptomatic) | 10 days | 10 days | 10 days | 10 days | 10 days |
| Staff Isolation Period (Asymptomatic) | 7 days | 7 days | 7 days (If negative on the 5th day, return on the 6th day) | 10 days | 10 days |
| Criteria for close contact with staff from COVID-19 patients | Regardless of the distance, cumulative contact of 15 minutes or more Starting point: 2 days before onset Patient: Regardless of mask usage Staff: If surgical mask or goggles were not worn | Contact with the maskless patient within 1 m for 15 minutes or more Starting point: 2 days before onset Patient: Without mask Staff: If surgical mask or goggles were not worn | Contact with patient within 1 m for 15 minutes or more Starting point: 2 days before onset Patient: Without mask Staff: If surgical mask or goggles were not worn | Contact with the maskless patient within 1 m for 15 minutes or more Starting point: 2 days before onset Patient: Without mask Staff: If surgical mask or goggles were not worn | Regardless of the distance and cumulative contact time If the patient is maskless Starting point: 2 days before onset Patient: Without mask Staff: If surgical mask or goggles were not worn |
| Criteria for close contact with staff during aerosol procedures | No regulation | No regulation | Regardless of the distance and cumulative contact time, contact distance within 2 m during the aerosol-generating procedure Starting point: 2 days before onset Patient: Without mask Staff: If N95 mask or goggles were not worn | Regardless of the distance and cumulative contact time and distance, during the aerosol-generating procedure Starting point: Date of onset Patient: Regardless of mask usage Staff: If N95 mask were not worn | Regardless of the distance and cumulative contact time and distance, during the aerosol-generating procedure Starting point: Date of onset Patient: Regardless of mask usage Staff: If N95 mask were not worn |
| Isolation period for close contact staff | 7 days after the last exposure | 7 days after the last exposure | 7 days after the last exposure | 7 days after the last exposure | Can return to work if negative on 5 days after last exposure |
| Conditions to ease isolation for staff with COVID-19 family members | 7 days after the last contact  Can return to work if negative on the 5th day PCR test Can return to work on 3rd day if negative on 2nd day PCR test, confirmed negative every day until the 5th day | 7 days after the last contact Can return to work if negative on the 5th day antigen test twice (4th and the 5th day) Can return to work after 10 days of health monitoring and negative antigen test | 7 days after the last contact Can return to work if negative on the 5th day after last exposure | 7 days after the last contact Can return to work after 10 days of health monitoring and negative PCR test | Can return to work if negative on 5 days after last exposure |
| Criteria for close contact with patients from COVID-19 staff | Contact with the maskless patient within 1 m for 15 minutes or more per day Starting point: 2 days before onset Patient: Without mask | Contact with the maskless patient within 1 m for 15 minutes or more per session Starting point: 2 days before onset Patient: Without mask | Regardless of cumulative contact time, distance within 1 m during conversation, procedure, or care Starting point: 2 days before onset Patient: Without mask | Regardless of the distance, cumulative contact of 15 minutes or more per session Starting point: Date of onset Patient: Without mask | Regardless of the distance and cumulative contact time Starting point: 2 days before onset Patient: Without mask |
| Isolation period for close contact patients | 5 days after the last exposure (until Aug 31, 2022)  6 days after the last exposure (From Sep 1, 2022) | 7 days after the last exposure | 7 days after the last exposure | 7 days after the last exposure | 7 days after the last exposure |
| AGMC, Amagasaki General Medical Center; AMC, Awaji Medical Center; HGMC, Harima-Himeji General Medical Center; KMC, Kakogawa Medical Center; TMC, Tamba Medical Center; PCR, polymerase chain reaction.  * The number of single-bed rooms eligible for special additional payments to hospitals for severe patient treatment environments and those for which patients are charged an extra room fee, reported by each medical institution to the Regional Bureau of Health and Welfare Director and other relevant authorities. The proportion was calculated by dividing the number of single-bed rooms by the total number of beds.  ^†^Antigen tests were used during hospital operating hours, and PCR tests were used outside of operating hours.  ^‡^ Liat SARS-CoV-2 was used since Aug 2022  ^§^PCR tests were used during hospital operating hours, and antigen tests were used outside of operating hours. | | | | | |


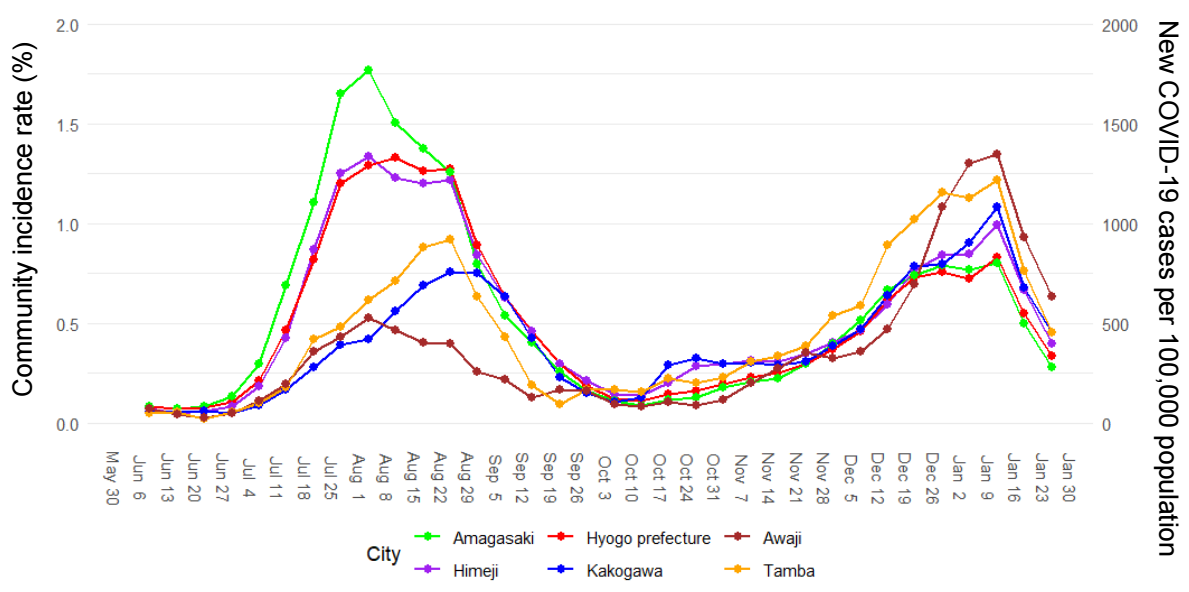


**Figure A.** Line graph of the weekly local COVID-19 incidence in Hyogo Prefecture and the cities where the participating medical institutions were located during the study period. The incidence was calculated by dividing the 7-day total number of new COVID-19 cases in Hyogo Prefecture and each city by the population of the prefecture or each city. The left vertical axis represents the percentage, while the right vertical axis shows the new case level per 100,000 population. In the first week of August, the weekly prevalence in Amagasaki City reached its peak at 1.77%.

**
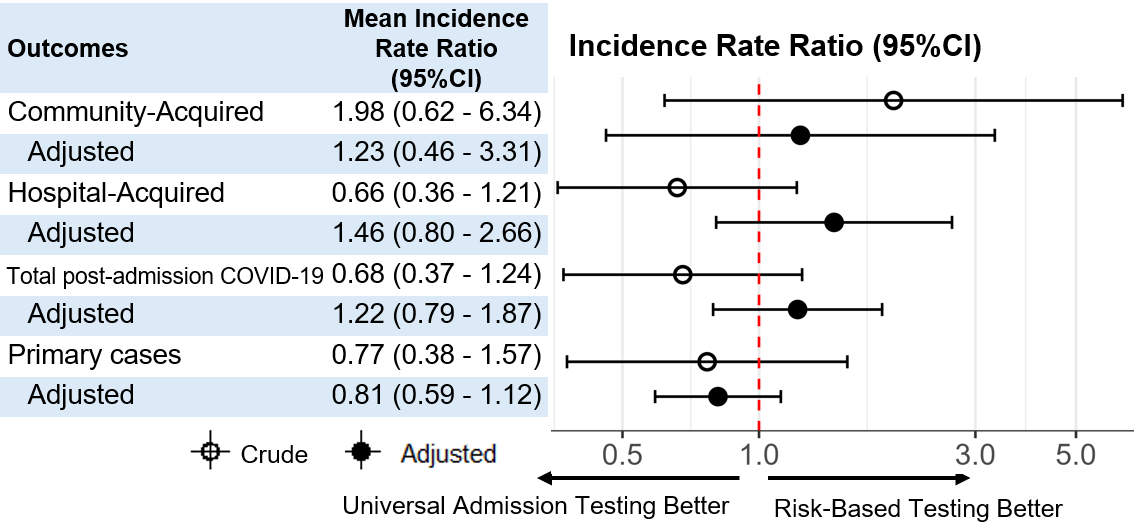
**

**Figure B.** Forest plot of the mean incidence rate ratio and 95% confidence intervals for each outcome. The white circles represent crude estimates, and the black circles represent adjusted estimates. A plot to the left indicates a lower risk with Universal admission testing, while a plot to the right indicates a lower risk with Risk-based testing. No significant risk reduction was observed for any outcome with Universal admission testing.

**
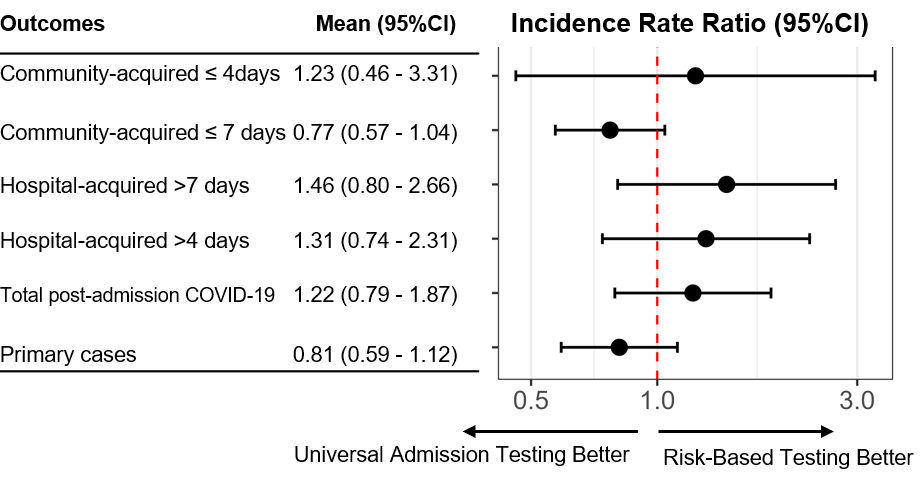
**

**Figure C.** Forest plot of the mean incidence rate ratio and 95% confidence intervals for each outcome, with changes to the cut-off for hospital-acquired and community-acquired cases. The cut-off for hospital-acquired cases was changed from 7 days to 4 days, and for community-acquired cases, from within 4 days to within 7 days. The white circles represent crude estimates, and the black circles represent adjusted estimates. A plot to the left indicates a lower risk with Universal admission testing, while a plot to the right indicates a lower risk with Risk-based testing.
